# The clinical effectiveness of intra-articular corticosteroids for arthritis of the lower limb in juvenile idiopathic arthritis: a systematic review

**DOI:** 10.1186/1546-0096-12-23

**Published:** 2014-06-11

**Authors:** Heidi Jennings, Kym Hennessy, Gordon J Hendry

**Affiliations:** 1 School of Science & Health, University of Western Sydney, Penrith, NSW, Australia; 2 Institute for Applied Health Research/School of Health and Life Sciences, Glasgow Caledonian University, Glasgow G4 0BA, UK

**Keywords:** Juvenile idiopathic arthritis, Intra-articular injection, Steroid injection, Systematic review, Lower limb, Knee, Ankle, Foot

## Abstract

**Background:**

Juvenile Idiopathic Arthritis (JIA) commonly affects joints of the lower limb including the knee, ankle, subtalar and other foot joints. Intra-articular corticosteroid injections (IACIs) are considered to be effective for short-term relief of synovitis, however, there appears to be a significant lack of published evidence from comparative effectiveness studies. The aim of this study was to identify and critically appraise the evidence for the efficacy of lower limb IACIs in children/adolescents with JIA.

**Methods:**

Studies were identified in databases Medline, Embase, CINAHL, AMED, PEDro, the Cochrane Library and TRIP, with no date restrictions. The primary search terms ‘juvenile idiopathic arthritis’, ‘lower limb’, ‘knee’; ‘ankle’, ‘foot’ and ‘intra-articular steroid injections’ and related synonyms were used to develop a comprehensive pragmatic literature search strategy. Included studies were quantitative longitudinal design such as randomised controlled trials, pseudo-randomised and non-randomised experimental studies, cohort studies, and case-control studies. All outcomes measures were subject to analysis. Quality assessment was conducted using the Cochrane Collaboration criteria with additional criteria for sample population representativeness, quality of statistical analysis and compliant intervention use and presence of co-interventions. Qualitative data synthesis was conducted for the outcome domains. Meta-analyses were not possible as multiple randomised controlled trials for outcome measures were not available. Levels of evidence were assigned to each outcome measure.

**Results:**

The inclusion criteria were met by twenty-one studies. One study had high quality for internal validity and nine studies had high quality for external validity. No studies had high quality for both internal and external validity. Four outcome domains were identified. There was weak evidence for IACIs decreasing clinical signs and symptoms in the lower leg, improving joint range of motion, decreasing leg length discrepancy, and for imaging techniques detecting the effects of IACIs.

**Conclusions:**

There is some weak evidence for the efficacy of IACIs improving certain outcome measures. However, there is also some inconclusive evidence due to a lack of quality studies. More high quality evidence is necessary to definitely determine the efficacy of IACIs for JIA in the lower leg.

## Background

Juvenile Idiopathic Arthritis (JIA) is the most common rheumatic disorder in children, affecting approximately 1 in 1000 Australian children [1]. Lower limb arthritis is common in JIA regardless of disease classification, with the knee joint affected most frequently at disease onset (40-60% of cases) followed by the ankle (20-30% of cases) [2,3]. Persistent synovitis in these joints is associated with pain, reduced joint ranges of motion, deformities, gait disruption, and poor functional status [4-7]. Moreover, radiographic progression of the disease occurs early, and if not addressed may result in permanent joint destruction and poor functional outcomes [8,9].

The primary aim of medical management in JIA is to suppress inflammation prior to the development of irreparable joint damage and functional limitation [10]. Recent improvements in the medical management of JIA have led to a greater emphasis on early diagnosis of JIA, early detection of synovitis, and subsequent prompt and aggressive systemic medical intervention via disease-modifying and/or biologic regimens to control the inflammatory disease process [11]. The introduction of such regimens have led to a relative increase in the number of patients achieving disease improvements characterised by low disease activity or remission [12]. However, in spite of promising signs of improvement, children may experience refractory disease, characterised by continued persistent synovitis, and/or serious adverse effects including hepato-toxicity and immuno-deficiency following administration of methotrexate and etanercept [13].

Intra-articular corticosteroid injections (IACIs) are often used as the main form of management in milder oligoarticular JIA, and also as an adjunct to systemic therapy in other JIA subtypes where localised synovitis is restricted to a single or a small number of joints [13,14]. IACIs are administered to induce rapid relief of symptoms through resolution of localised synovitis, and in some cases may prevent the need for escalation of systemic therapy and associated increased risk of serious side-effects [14]. It is acknowledged that IACIs are also associated with adverse events, however, these may be relatively mild compared to those associated with systemic disease-modifying anti-rheumatic drugs (DMARDs) or biologics. These may include subcutaneous atrophy, hypopigmentation and/or post-injection pain [14,15]. Several inconsistencies have been highlighted in studies of IACIs such as variable injection techniques, inclusion criteria, post-injection procedures, outcome measures, and follow-up period [14,16,17].

Previous systematic reviews have been conducted but they are now outdated and new studies of IACIs have been published since they were completed. The Cochrane Collaboration recommends that systematic reviews are updated biannually, and the most relevant and recent review was published seven years ago [18]. Two systematic reviews have been conducted to evaluate the evidence for IACIs in JIA; one which focused on IACIs for temporomandibular joint (TMJ) arthritis only [16]; and another which reviewed studies on lower limb IACIs for children with JIA and/or adults with rheumatoid arthritis (RA). However, ultimately they did not include any studies of JIA [17]. The recent systematic review of IACIs for TMJ arthritis [16] may not be directly relevant to this work as IACIs administered in the joints of the lower limb appear to be less efficacious than those administered in upper limb and non-weight-bearing joints [19]. Moreover IACIs administered to the knee joints appear to be more efficacious than those administered to the small joints in the foot, where the response is less predictable [19]. Two other reviews of the overall medical management of JIA adopted systematic approaches to conduct their literature search, but their restrictive inclusion criteria retrieved only two small randomised studies concerning IACIs and only limited conclusions were drawn [10,20]. One other systematic review attempted to appraise the evidence for knee joint IACIs for a range of arthritis conditions including JIA, osteoarthritis (OA) and RA [21]. However, methodological problems were identified with this review including a single joint focus (knee), adult arthritis predominance for studies included, and violations of the detailed inclusion criteria. Narrative reviews have also been conducted and have provided useful summaries of the literature concerning IACIs in JIA [14,22]. However, such reviews are often reinforced with the authors’ expert opinions and clinical experience and as a result may be vulnerable to bias [23].

Most research concerning IACIs efficacy to date appears to focus predominantly on the knee joint only, or a combination of several joints which differ in terms of anatomical complexity, and may include weight-bearing/non-weight-bearing joints which could impact on IACI efficacy. The reviews which have been published to date fail to account for the efficacy of IACIs for synovitis relief in other important lower limb joints, and thus the findings and conclusions drawn from these reviews are not necessarily generalizable. Lastly, it should be noted that standard clinical practice has changed in recent years with a greater emphasis being placed on image-guidance to aid the administration of intra-articular injection therapies [24-26]. This advancement of clinical practice may have led to improvements in the effectiveness of IACIs on the basis of improved steroid placement accuracy and thus fewer reports of adverse events [24-26]. The emergence of further evidence, introduction of new technologies and changing clinical practices has raised important new questions concerning lower limb IACI efficacy in JIA. Accordingly, the aim of this review is to systematically identify and critically evaluate the clinical effectiveness of IACIs administered to joints of the lower limb in people with JIA.

## Methods

### Search strategy

A detailed electronic database search of the literature was performed using the following databases: Medline, Embase, CINAHL, AMED, PEDro, the Cochrane Library, and TRIP. Reference lists of eligible studies identified by the electronic database search were searched by hand to identify studies not initially found in the electronic search. Personal reference lists of the reviewers were also hand searched to identify any studies known to the reviewers that may not have been identified by the initial search strategy. The following search terms ‘juvenile idiopathic arthritis’, ‘lower limb’, ‘knee’, ‘ankle’, ‘foot’, and ‘intra-articular steroid injections’ and related synonyms were used to develop a comprehensive pragmatic literature search strategy. Standard MeSH terms were utilised where possible or an appropriate text word was adopted. Boolean operators (such as ‘explode’, ‘OR’, ‘*’, ‘$’ and ‘AND’) were used. The full search strategy is outlined in Additional file 1.

### Study inclusion criteria

Studies included were of a quantitative longitudinal design, such as randomised controlled trials (RCTs), pseudo-randomised and non-randomised experimental studies, cohort studies, and case-control studies. Studies of single cases were excluded. Studies reporting children, adolescents and adults who have JIA were included. Studies of IACIs as the single primary intervention, in combination with systemic medications, and/or in combination with non-pharmacological interventions were included. All outcome measures/variables were subject to analysis. Literature was limited to full text published articles available in English due to language barriers of both independent reviewers. No restriction was imposed on year of publication.

Study abstracts found from the electronic database search and by hand searching were reviewed for eligibility based upon the above inclusion criteria. Two independent reviewers (HJ and KH) selected abstracts which appeared to meet the inclusion criteria. Full text articles of the selected abstracts were obtained and compared against the inclusion criteria. The full text, original research articles meeting the inclusion criteria were assessed for quality.

### Quality assessment

Quality assessment was conducted by two independent reviewers (HJ and KH) using an adaption of The Cochrane Collaboration tool for assessing risk of bias as previously described [18,27]. For randomised studies the internal validity criteria included sequence generation, allocation concealment, blinding of participants and personnel, blinding of outcome assessment, incomplete outcome data, selective outcome reporting, and selective outcome reporting. Whereas for non-randomised studies, adapted criteria were adopted that did not include criteria that was relevant to randomised studies only. Further internal validity criteria were also included to determine presence of co-interventions, intervention compliance, and if proper statistical analysis was conducted. External validity criteria were also included to determine restrictiveness of the inclusion and exclusion criteria, and to determine if the sample population was representative of the general population with JIA. For a study to achieve a high quality score overall, all included domains had to be scored independently as high quality by both reviewers. A study that was independently rated with ≥1 domain scored as low quality was rated as low quality overall. Any disagreement was resolved by a third reviewer (GJH).

### Data extraction/evidence grading

The data extraction/evidence grading system was agreed upon by the co-authors *a priori* and is outlined as follows. Due to a lack of multiple RCTs for specific outcomes measures, meta-analysis was not possible and as such qualitative data synthesis was conducted. Once extracted data was analysed and synthesised, an evidence rating was assigned according to previously published criteria [28] (Table 1). Once studies were rated for quality, they were grouped according to the outcomes measured and then the evidence grading system was adopted to assign levels of evidence for each outcome. This evidence grading criteria considers both quality and quantity of studies as well as the consistency of the findings for each outcome [28].

**Table 1:** **Evidence rating criteria (adapted from Ariens et al, 2000 **[28]**)**

**Strong**	**At least 2 studies of high quality with consistent findings (agreement of >75% of studies)**
Moderate	1 high quality study and at least 2 low-quality studies with consistent findings (agreement of >75% of studies)
Weak	At least 2 low-quality studies with consistent findings (agreement of >75% of studies)
Inconclusive	Insufficient and/or conflicting studies

## Results

**Figure 1:**
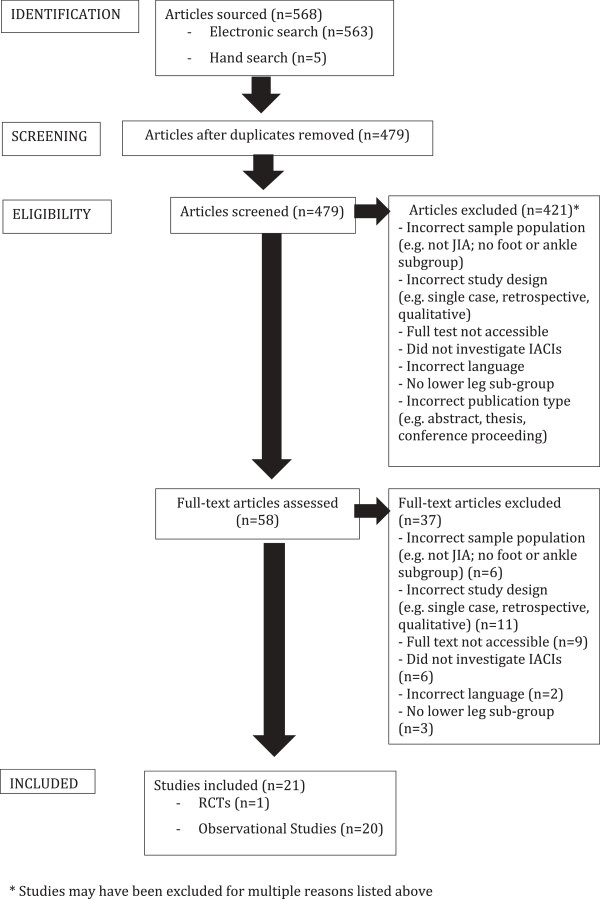
**Flow chart of literature search for IACI use in children with JIA in the lower limb (PRISMA diagram adapted from Moher et al, 2009**[29]**).**

Using the detailed search strategy, a total of 568 articles were retrieved. The inclusion criteria were met by twenty-one studies (see Figure 1), and a description of each study is presented in Table 2. The majority of the studies were observational in nature, with only one RCT identified for inclusion. Therefore, meta-analyses were not possible as were originally intended. One study had high quality internal validity and twenty had low quality internal validity. Nine studies had high quality external validity and twelve had low quality external validity. There were no studies with both high quality internal and external validity (Table 3). From the assessed studies, nine outcome domains were identified which included tenderness/pain, swelling, synovitis, effusion, hyperthermia, sustained ‘clinical response’, joint range of motion (ROM), length discrepancy (leg length discrepancy or joint circumference), and imaging detectable outcomes. Leg length discrepancy (LLD) was defined as unequal length of the lower limb from the knee distally, as a result of knee synovitis and/or tibial growth disturbances. Qualitative syntheses of results are outlined below and presented in Table 4.

**Table 2:** Description of included studies

**Author, year**	**Study type**	**Participant description**	**No. entered/ completed study**	**Follow-up period**	**Intervention (including post-injection procedure)**	**Outcome measures**
Balogh et al, [30]	RCT	Fulfilment of EULAR/WHO Oslo Criteria for JCA.	23/23 (23 knees)	1, 3, 7 and 42 days	TH in 11/23	Knee Joint Circumference (cm); Knee Joint Flexion (degrees).
					BM in 12/23	
					(Dose not recorded)	
		Pauciarticular form of disease.			Post-injection: Not specified.	
		Co-interventions: Not specified				
Al-wahadneh, [31]	OBS	Failure to respond to NSAIDs with/without slow acting anti-inflammatories.	24/24 (30 knee joints)	3, 6, 9, 12 and 24 months.	MA 1 mg/kg/joint mixed with 1 cc 1% lidocaine without adrenaline.	Sustained Clinical Response: ‘Active Inflammation’ (joint effusion and heat and tenderness/pain with/without correction of deformity)
		Co-interventions: 10/24: MTX (10 mg/kg/wk), 24/24: Naproxen (10-20 mg/kg/day), 24/24: NSAIDs, 11/24: Prednisolone (0.25 mg/kg/day)			Post-injection: Immobilisation 24 hrs post injection before commencing physiotherapy.	
Allen et al., [32]	OBS	<16 yrs of age at onset of chronic arthritis 29/40 pauciarticular onset JRA	40/40 (53 knees) 4 patients lost to follow-up.	3, 6, 12 and 24 months.	20-40 mg TH with 1% xylocaine without epinephrine infiltration.	Sustained Clinical Response: ‘Active
		4/40 seronegative enthesopathy-arthropathy syndrome			Post-injection: Not specified	Inflammation’(Joint effusion and heat and tenderness, with or without complete correction of deformity)
		6/40 psoriatic arthritis				
		Co-interventions: 100%- acetylsalicylic acid for at least 3 months; 30%- 1 NSAID; 22.5%- 2 NSAIDs; 20%- ≥ 3 NSAIDs; 3 patients previously on oral prednisone; 1 patient on hydroxychloroquine; 4 patients prior corticosteroid injection (other than TH); all patients maintained on the same NSAIDs post-injection.				
Beukelman et al., [26]	OBS	Definitive JIA diagnosis based on criteria.	38/38 (55 STJ injections)	1-30 weeks (median = 6 weeks)	TH in 13/38TA in 24/38	Sustained Clinical Response: STJ Eversion/Inversion and pain and gait abnormalities
		Decreased foot inversion or eversion on physical examination			(Medication and dose not recorded in 1/39 injection)	
		Co-interventions: 14/38: MTX, 3/38: TNF-alpha inhibitor, 3/38: TNF-alpha inhibitor and MTX.			TA used when TH was commercially unavailable.	
					Post-injection: Non-weight bearing 24 hrs post-injection. Normal activity resumed afterwards.	
Cahill et al., [25]	OBS	Clinical signs of STJ inflammation	38/38; 24/38	2-3 months	0.5-1 ml, 20 mg/ml TH	Increased STJ
		Referral for image-guided IACI.	single STJ; 4/38 bilateral STJ; 3/38 subsequent contra-lateral STJ; 7/38 at least one repeated STJ.	(active disease) 6 months (without active disease)	Post-injection: Not specified.	inversion and eversion: Normal ROM, without pain and limping
		Co-interventions: Not specified.				
Earley et al., [33]	OBS	Pauci-articular onset JCA.	23/23 (86 knees)	3, 6 and 12 months	TH 20 mg for children weighing <20 kg (63 knees) OR for children >20 mg 40 mg (20 knees)	Sustained Clinical Response: Soft tissue swelling and joint effusion and degree of flexion contracture and degree of valgus deformity.
		Painful swollen knee with poor function.				
		Not respondent to at least 3 months of conventional treatment.				
					Post-injection: Not specified.	
		Co-interventions: All patients were on at least one NSAID and physiotherapy and splinting, 3/23 on gold, 1/23 on prednisolone				
Eberhard et al., [34]	OBS	JRA diagnosis based on ACR criteria.	85/85 (51/99 received TH and 48/99 received TA; 14 patients received both)	2 weeks then every 3 months for a minimum for 15 months.	TH: 40 mg (knee), 30 mg (ankle).	Sustained Clinical Response: Non-bony swelling and (if no swelling) limitation ROM pain on motion or joint tenderness.
		Co-interventions: TH group: 33/51 NSAIDs; 16/51 MTX; 9/51 sulfasalzine; 3/51 prednisone + etanercept; 14/51 no medication.				
					TA: 80 mg (knee), 60 mg (ankles)	
					Post injection: Minimal activity for 24 hrs post-injection.	
		TA group: 33/51 NSAIDs; 12/51 MTX; 2/51 sulfasalazine; 4/51 etanercept; 2/51 prednisone; 2/51 leflunomide; 15/51 no medication.				
Eich et al., [35]	OBS	JCA diagnosis based on EULAR criteria.	15/15 (11 knees)	Clinical and US assessment: 1 week and 1 month MRI: 1 month	TH: 40 mg (knee)	Pain
					Post-injection: Not specified.	
		Failure of systemic therapy and physiotherapy.				Swelling
						Hyperthermia
		Local growth disturbances.				
						Limited ROM
		Popliteal cyst in affected knee.				
						Leg-length discrepancy
		With/without complete deformity correction.				
						MRI: Joint effusion
		Co-interventions: Not specified.				
						Popliteal cyst
						Destruction of articular cartilage and/or bone
						Destruction of menisci
						Marrow oedema
						Avascular necrosis
						US: Joint effusion and/or pannus
						Popliteal cyst
Hertzberger-ten Cate et al., [36]	OBS	Type 1 pauciarticular JCA.	21/21 (27 knees)	6 months	TA: 20 mg in children weighing >20 kg with 1 ml lignocaine.	Sustained Clinical Response: swelling and synovial fluid and no increased temperature.
		Chronic arthritis in ≥1 knee.				
		No response ≥6 months of conventional treatment.				
		Flexion contracture, muscle wasting and/or growth disturbances.				
		Co-interventions: 81% used splints at night and 1 hr during the day, 50% received physiotherapy, 77% received NSAIDs.			Post-injection: knee passively flexed and extended several times to distribute drug. No advice regarding activity levels.	
Honkanen et al., [37]	OBS	JIA diagnosis.	79/79 (79 knees)	6-8 weeks	MA: in 45/79 (Mean dose 1.5 mg/kg)	Sustained Clinical Response: absence/presence of ‘symptoms’
		With/without previous IACI.				
		Co-interventions: 100% on NSAID agents and regular physiotherapy, 17/79 on hydroxychloroquine, sodium aurothiomalate or auranofin (slow-acting antirheumatics), 5/79 on alternate day glucocorticoid therapy.			TH: in 34/79 (mean dose 0.7 mg/kg)	
					Post-injection: Non- weight bearing for 24 hrs.	
Huppertz et al., [38]	OBS	Children with chronic arthritis, not responding to NSAIDs.	21/21(18 knees, 2 ankles)	7, 13 weeks	Knees: 1 mg/kg TH with 20 mg min dose and 60 mg maximum dose.	Joint Swelling
		Co-interventions: Not pre-specified. After 13 weeks, 10/21 had been treated with concomitant NSAIDs and 5/21 received chloroquine.				Effusion
					Ankle: “a lower dose”	
					Post injection: Not specified.	Joint limitation
						Joint tenderness/pain
Laurell et al., [24]	OBS	JIA diagnosis based on ILR criteria.	30/30 (40 ankle regions)	4 weeks	TA 40 mg/ml	Pain
					Post-injection: Not specified.	US: Synovial hypertrophy Synovial
		Active disease.				
		Co-interventions: 26 patients had ongoing systemic treatment: 58% with MTX				hyperaemia
		23% with MTX and biologics (3 etanercept, 2 adalimumab, 1 abatacept), 19% with systemic corticosteroids				Joint ROM
Lepore et al., [39]	OBS	JIA patients.	37/37 (87 injections of 37 knees)	7-65 months (average of 31.3 months)	TH: 1 mg/kg (maximum 40 mg)	Sustained Clinical Response: ‘Clinical signs of inflammation’
		Failure to respond to 2 months NSAIDs.				
					Post-injection: Advised to keep child at home for first 24 hrs and to avoid physical exertion and carrying weights.	
		Relapse after full remission period.				
		Co-interventions: NSAID use was discontinued in all patients at time of local treatment				
Marti et al., [19]	OBS	JIA patients who received injections and follow up as in patients.	60/60 (108 knees; 29 ankles, 5 STJ and 3 midfoot)	Knee: 1-69 months, Ankle: 1-39 months, STJ: 13 months, Midfoot: 0-3months. Digits: 18-66 months	TH: 40 mg (knee, shoulder and hips); 20 mg (wrist, elbow, ankle and STJ); 5 mg (finger and toes)	Sustained Clinical Response: Swelling and effusion and tenderness/pain
		Co-interventions: Non-steroidal anti-rheumatic drugs, MTX, Systemic corticosteroids, Salazopyrine				
					TA: 80 mg (knee, shoulder and hips); 40 mg (wrist, elbow, ankle and STJ); 10 mg (finger and toes).	
					Children with a body weight 20-40 kg received 75% of these doses. Children with body weight <20 kg received 50% of these doses.	
					Post-injection: Advised to keep injected joint as quiet as possible for 24 hrs post-injection.	
Papadopoulou et al., [40]	OBS	Diagnosis JIA based on ILR criteria.	220/220 (186 knees, 168 ankles, 67 STJ, 14 MTJ and 14 IPJ)	6 months	TH: 1 mg/kg (maximum 40 mg) in knee & hips; 0.75 mg/kg (maximum 30 mg) in ankles.	Synovitis
					MA: 20-40 mg in STJ & intertarsal joints; 5-10 mg in smaller foot joints.	
					Post-injection: Avoid activity or weight bearing for 24 hrs post-injection.	
		Previous IACIs with minimum follow-up of 6 months.				
		Co-interventions: 61.8% of patients received systemic medications including: MTX (56.8%),				
		Biologic agents (9.5%), Systemic corticosteroids (11.4%)				
Ravelli et al., [41]	OBS	JIA diagnosis.	94/94 (66/94- unilateral knee 28/94 bilateral knees)	6 months	TH: 1 mg/kg (maximum 40 mg) with 0.5 ml lignocaine (2%).	Sustained Clinical Response: Synovitis
		Initial injection between Feb 1996 and June 1990.				
		Co-interventions: 57% on NSAIDs, 24% on NSAIDs and “2nd line drugs” (no specification of second line drugs)				
					Post-injection: Rest joints for 24 hrs	
Remedios et al., [42]	OBS	Children with JIA presenting as painful swollen ankles.	11/11 (13 ankles)	>64 weeks	TH: 20 mg with 1 ml 0.5% bupivacaine.	Sustained Clinical Response: Synovitis (clinically assessed)
		Co-interventions: Not Specified			Post-injection: Not specified.	MRI: Pannus
Sherry et al., [43]	OBS	Pauciarticular JCA from ACR criteria.	16/16 (15 knees and 6 ankles)	Mean follow-up University of Washington group: 42 months (SD +/- 11).	University of Washington Group: 20 mg TH within 2 months of diagnosis.	Leg Length Discrepancy (cm)
		<7 years at diagnosis.				
		Reviewed at rheumatology centres at University of Washington or North Carolina (University of North Carolina/Duke University)				
				North Carolina group: 46 months (SD +/- 15)	North Carolina Group: No IACI.	Thigh circumference discrepancy (cm)
		Co-interventions: University of Washington Group: 25% on DMARD therapy, 44% with physical therapy evaluation, 31% with splints, 0% with shoe lifts North Carolina Group: 21% on DMARD therapy, 57% with physical therapy evaluation, 43% with splints, 50% with shoe lifts			Post-injection: Not specified.	
Sornay-Soares et al., [44]	OBS	Children meeting the 1997 Durban criteria for JIA with knee involvement.	8/8 (13 knees)	6 months, 12 months	Joint lavage using 2 needles and 0.5-1.51 ml saline. Followed by one vial of TH, except in 2 knees (one patient) BM was used.	Sustained Clinical Response: Pain and joint effusion
		Co-interventions: 6/8 on NSAIDs, 5/8 on MTX, 1/8 on Azathioprine, 2/8 on Cyclosporine				
					Post-injection: Knees were taped and advised to rest for 24 hrs, keeping in extension with walking crutches. Ice packs could be used for pain relief.	
Verma et al., [45]	OBS	Diagnosis of unresponsive oligoarticular/polyarticular JIA.	13/13 (13 knees and 3 ankles) 3 patients were lost to follow-up at 6 months due to uncontrolled arthritis.	6, 12 weeks.	TA (0.5-1 ml, 20-40 mg).	Mid-leg circumference (cm)
		Joint swelling/effusion, limitation of ROM, tenderness, pain, warmth.	4 children were lost to follow-up at 12 months.		Post-injection: Reduced movement for 24 hrs.	
		12 weeks daily oral naproxen and/or weekly MTX.				
		Co-interventions: All patients were on NSAIDs.				
Zulian et al., [46]	OBS	Diagnosis persistent or extended oligoarticular JIA	85/85 (115 knees and 15 ankles)	1, 3, 6, 9, 12, 18 and 24 months	TH: 42 patients treated; 1 mg/kg (>40 mg)	Sustained Clinical Response: Swelling and limited ROM and pain and warmth.
		Patients managed at University of Padua paediatric rheumatology unit			TA:43 patients treated; 1 mg/kg (>40 mg) (Availability issues of TH meant TA was used as an alternative in some cases).	
		Received IACIs from Jan 1996 and Dec 2000.				
		Unsatisfactory response to NSAIDs.				
		Persistent isolated joint involvement.			Post-injection: Non-weight bearing for	
		Co-interventions: TH: 64.3% on NSAIDs, 11.4% on MTX			at last 72 hrs post-injection.	
		TA:51.7% on NSAIDs, 5% on MTX				

OB = observational; RCT = randomised controlled trial; JIA = juvenile idiopathic arthritis; JCA = juvenile chronic arthritis; JRA = juvenile rheumatoid arthritis; WHO = World Health Organization; ACR = American College of Rheumatology; EULAR = European League Against Rheumatism; ILR = International League of Associations for Rheumatology; ROM = range of motion; MTX = methotrexate; IACI(s) = intra-articular corticosteroid injection; TH = triamcinolone hexacetonide; TA = triamcinolone acetonide; NSAID(s) = Non-steroidal Anti-Inflammatory Drug(s); MA = methylprednisolone acetonide; BM = betamethasone; IACI(s) = intra-articular corticosteroid injection(s); US = ultrasound; MRI = magnetic imaging resonance; STJ = subtalar joint; MTPJ = metatarsophalangeal joint; IPJ = interphalangeal joint; SD = standard deviation; TNF-alpha = tumour necrosis factor alpha; DMARD = Disease Modifying Anti-Rheumatic Drug.

**Table 3:** Quality assessment of included studies

**Author**	**Sequence generation/allocation concealment (internal validity)**	**Blinding of participants, personnel and outcome assessors (internal validity)**	**Incomplete outcome data (internal validity)**	**Selective outcome reporting/statistical issues (internal validity)**	**Interventions (internal validity)**	**Generalisability (external validity)**	**Internal validity**	**External validity**
Balogh et al., [30]	Low	Low	Low	High	Low	Low	Low	Low
Al-wahadneh, [31]	N/A	N/A	Low	Low	High	Low	Low	Low
Allen et al., [32]	N/A	N/A	Low	Low	Low	High	Low	High
Beukelman et al., [26]	N/A	N/A	Low	Low	High	High	Low	High
Cahill et al., [25]	N/A	N/A	Low	Low	Low	Low	Low	Low
Earley et al., [33]	N/A	N/A	Low	Low	High	Low	Low	Low
Eberhard et al., [34]	N/A	N/A	Low	Low	High	High	Low	High
Eich et al., [35]	N/A	N/A	High	Low	Low	High	Low	High
Hertzberger-ten Cate et al., [36]	N/A	N/A	Low	Low	High	Low	Low	Low
Honkanen et al., [37]	N/A	N/A	Low	Low	High	Low	Low	Low
Huppertz et al., [38]	N/A	N/A	High	Low	Low	Low	Low	Low
Laurell et al., [24]	N/A	N/A	Low	Low	Low	High	Low	High
Lepore et al., [39]	N/A	N/A	Low	Low	Low	Low	Low	Low
Marti et al., [19]	N/A	N/A	Low	Low	Low	High	Low	High
Papadopoulou et al., [40]	N/A	N/A	Low	High	High	High	Low	High
Ravelli et al., [41]	N/A	N/A	Low	Low	Low	High	Low	High
Remedios et al., [42]	N/A	N/A	Low	Low	Low	Low	Low	Low
Sherry et al., [43]	N/A	N/A	Low	High	High	Low	Low	Low
Sornay-Soares et al., [44]	N/A	N/A	Low	Low	High	High	Low	High
Verma et al., [45]	N/A	N/A	Low	Low	Low	Low	Low	Low
Zulian et al., [46]	N/A	N/A	High	High	High	Low	High	Low

**Table 4:** Qualitative synthesis of results and overview of evidence

**Domain**	**Author, year (ref.)**	**Results**	**Adverse effects**	**Level of evidence (specific areas)**	**Level of evidence (for domain)**
Tenderness/pain	Eich et al, [35]	Knee: 0% after 1 month (no significance value given)	One patient experienced focal cutaneous atrophy and a possible ruptured popliteal cyst	Weak evidence for IACIs decreasing pain in the knee as 2 studies show a reduction of pain	Weak evidence for IACIs decreasing pain in lower leg joints overall as 3 studies show a reduction of pain
	Huppertz et al, [38]	Knee: 33.3% after 7 weeks (no significance value given)	Not Specified		
	Laurell et al, [24]	Ankle: Pain regression/partial improvement = 92.5% (no specific data included, only percentages) after 4 weeks	Local subcutaneous atrophy in 3 patients at 4 injection sites	Inconclusive due to lack of studies	
		(No significance value given)			
Swelling	Eich et al, [35]	Knee: 27.3% after 1 month (no significance value given)	One patient experienced focal cutaneous atrophy and a possible ruptured popliteal cyst		Weak evidence for IACIs decreasing swelling in lower leg joints overall as 2 studies show a reduction in swelling
	Huppertz et al, [38]	Knee: 0% after 7 weeks (no significance value given)	Not Specified		
Synovitis	Papadopoulou et al., [40]	Number of joints in remission vs.	0.9% of injected joints suffered from skin hypopigmentation or subcutaneous atrophy.	Weak evidence for IACIs decreasing synovitis in the knee, ankle and STJ as 2 studies for each joint showed a reduction of synovitis.	Weak evidence for IACIs decreasing synovitis in lower leg joints overall as 3 studies show reduction of synovitis
		Number of joints with synovitis flare: Knee = 79.2% vs. 20.8% (p < 0.001); Ankle = 54.8% vs. 45.2% (p = 0.14) STJ = 65.5% vs. 34.5% (p < 0.0001); MTPJ = 85.7% vs. 14.2% (p = 0.008); IPJ = 90.0% vs. 10.0% (p = 0.0003).			
			“A few” patients developed flushing or redness of the cheeks 24-48 hrs post-injection.		
				Inconclusive evidence for MTPJ and IPJ due to lack of studies	
		Overall mean relapse time 0.5 yrs (IQR 0.3-1.3 yrs) vs. mean remission time 0.9 yrs (IQR 0.6-1.9 yrs).			
	Ravelli et al., [41]	Knee: continued resolution at 6 months = 69%	One patient suffered from subcutaneous atrophy at the injection site.		
		Reoccurrence/relapse = 31%			
		(no significance value given)			
	Remedios et al., [42]	Clinical synovitis vs. MRI pannus:	Not Specified.		
		Tibiotalar: 84.6% vs. 84.6%; STJ: 23.1% (definite) vs. 46.2% (no significance value given)			
		Mean duration of effect (guided vs. unguided) (weeks): 38.3 vs. 13.9 (no significance value given)			
Effusion	Huppertz et al, [38]	Knee: 13.3% after 7 weeks (no significance value given)	Not Specified		Inconclusive due to lack of studies
Hyperthermia	Eich et al, [35]	Knee: 18.2% after 1 month (no significance value given)	One patient experienced focal cutaneous atrophy and a possible ruptured popliteal cyst		Inconclusive due to lack of studies
Sustained clinical response	Al-Wahadneh, [31]	Knee: 30/30 = 100% maintained resolution at 3 months (no significance value given)	1/24 (4%): short-lived pain and erythema, 2/24 (8%): subcutaneous atrophy resolved dramatically after one year, 2/24 (8%): asymptomatic periarticular calcification.	Weak evidence for IACIs decreasing clinical signs and symptoms in the knee, ankle and STJ as 11, 4 and 2 studies respectively showed sustained clinical response.	Weak evidence for IACIs decreasing clinical signs and symptoms in lower leg joints overall as 21 studies show sustained clinical response
				Inconclusive evidence for midfoot due to lack of studies	
	Allen et al, [35]	Knee: 18/48 = 37.5% relapse at 6 months (no significance value given)	Subcutaneous fat atrophy at injection site in one patient.		
	Beukelman et al, [26]	Knee: 1.4 yrs of resolution before relapse (SD ± 1.0)	53%: subcutaneous atrophy or hypopigmentation at injection site.		
		STJ: 1.2 yrs of resolution before relapse (TH + TA) (SD ± 0.9)			
	Earley et al, [33]	Knee: Excellent/Good Outcome: 92.8% vs.	2/23 had areas of subcutaneous atrophy at injection site.		
		Poor/Re-injected: 7.2% at 3 months (No significance value given)			
	Eberhard et al, [34]	Knee Median Relapse (months)	Not Specified.		
		TH: 11.1 +/- 0.81			
		TA: 7.95 +/- 0.95			
		(p = 0.0072)			
	Hertzberger-ten Cate et al, [36]	Knee: resolution maintained in 70% of knees for >6 months (No significance value given)	2/21 patients suffered a small atrophic lesion at the injection site.		
			1/21 patient suffered a red and painful knee the day following injection (resolved by local ice application).		
Sustained clinical response (cont’d)	Honkanen et al, [37]	Knee: overall probability of sustained clinical response was higher for TH then MP after 6-8 weeks (p > 0.0005)	Not Specified.		
	Laurell et al, [24]	Ankle pain Regression/Partial Improvement = 92.5% (no specific data included, only percentages) after 4 weeks (No significance value given)	Local subcutaneous atrophy in 3 patients at 4 injection sites.		
	Lepore et al, [39]	Knee: mean remission time = 13.9 months (range = 0-54 months) (no significance value given)	10% (3 patients) subcutaneous lipolysis with spontaneous regression.		
	Marti et al, [19]	Mean duration of remission until flare in months (range): knee = 8.0 (0-27); ankle = 4.5 (0-13); STJ = 3.5 (0-11); midfoot = 1 (0-3) (no significance value given)	Systemic effects of glucocorticoids in 7 patients (one flushed cheeks, three increased appetite, three mood changes).		
		Mean follow-up time of joints with ongoing remission in months (range):knee = 27.2 (1-69); ankle = 18.2 (1-39); STJ = 13; midfoot = n/a (no significance value given)	Local side effects in 12 patients (14 skin atrophies combined with hypopigmentation).		
	Remedios et al., [42]	Clinical synovitis vs. MRI pannus:	Not Specified.		
		Tibiotalar: 84.6% vs. 84.6%; STJ: 23.1% (definite) vs. 46.2% (no significance value given)			
		Mean duration of effect (guided vs. unguided) (weeks): 38.3 vs. 13.9 (no significance value given)			
Sustained clinical response (cont’d)	Sornay-Soares et al., [44]	Knee: continued resolution in 76.9% knees at 6 months	No adverse effects were recorded.		
		(no significance value given)			
	Zulian et al., [46]	Knee and Ankle: continued resolution at 6 months	2 patients in each group developed skin atrophy at the injection site.		
		TH: 81.4%	2 patients experienced reversible apnoea (<20secs duration) during the induction phase of sedation.		
		TA: 53.3%			
		(p = 0.001)			
Joint ROM	Balogh et al., [30]	Knee: BM = 0 degree difference vs. TH = 13 degrees difference at 42 days (no significance value given)	Mild skin atrophy in 1/23 who was injected with TH	Weak evidence for IACIs increasing ROM in the knee as 3 studies show improvement	Weak evidence for IACIs increasing ROM in lower leg joints overall as 5 studies show improvement
	Eich et al, [35]	Knee: 66.7% improvement after 1 month (no significance value given)	One patient experienced focal cutaneous atrophy and a possible ruptured popliteal cyst		
	Huppertz et al, [38]	Knee: 92.9% improvement after 7 weeks (no significance value given)	Not Specified		
	Laurell et al, [24]	Ankle: 95% improvement after 4 weeks	Local subcutaneous atrophy in 3 patients at 4 injection sites	Inconclusive due to lack of studies	
		(No significance value given)			
	Cahill et al., [25]	STJ: 89.5% returned to normal ROM within 13 weeks (no significance value given). Mean duration of improvement = 1.2 SD ±0.9 yrs.	Subcutaneous atrophy or hypopigmentation in 53%	Inconclusive due to lack of studies	
Leg length discrepancy	Eich et al, [35]	Improvement after 1 month: 100% (no significance value given)	One patient experienced focal cutaneous atrophy and a possible ruptured popliteal cyst		Weak evidence for IACIs decreasing LLD as 3 studies show reduction
	Sherry et al., [43]	Mean difference in leg length for early intervention vs. control:	Not Specified		
		0% (±0) vs. 1.0% (±1.4) (p = 0.005)			
		over mean follow up of 4 months (SD ± 11 months)			
	Verma et al., [45]	Mean Lower Leg Difference (cm): â†“ 0.22 at 6 and 12 weeks (no significance value given).	No adverse effects were recorded.		
Circumference	Balogh et al., [30]	Mean Knee Joint Circumference (cm):	Mild skin atrophy in 1/23 who was injected with TH		Inconclusive due to lack of studies
		BM = 1.0			
		TH = -1.7 after 1, 3, 7 and 42 days (no significance values given).			
Imaging					Weak evidence for IACIs decreasing imaging findings in lower leg joints overall as 3 studies show improvement
MR imaging					Weak evidence for IACIs decreasing MRI detectable clinical signs and symptoms as 3 studies show detectable improvement
Effusion	Eich et al., [35]	Knee: 36.4% detected by MRI at 1 week and 1 month (no significance value given)	One patient experienced focal cutaneous atrophy and a possible ruptured popliteal cyst	Weak evidence for IACIs decreasing MRI detectable effusion as 2 studies show detectable improvement.	
	Huppertz et al, [38]	Knee and Ankle: 40.0% at 7 and 13 weeks (no significance value given)	Not Specified.		
Pannus	Eich et al., [35]	Knee: 63.6% detected by MRI at 1 week and 1 month (no significance value given)	One patient experienced focal cutaneous atrophy and a possible ruptured popliteal cyst	Weak evidence for IACIs decreasing MRI detectable pannus as 3 studies show detectable improvement.	
	Huppertz et al, [38]	Knee and Ankle: 10.0% at 7 and 13 weeks (no significance value given)	Not Specified.		
	Remedios et al., [42]	Clinical synovitis vs. MRI pannus:	Not Specified.		
		Tibiotalar: 84.6% vs. 84.6%; STJ: 23.1% (definite) vs. 46.2% (no significance value given)			
		Mean duration of effect (guided vs. unguided) (weeks): 38.3 vs. 13.9 (no significance value given)			
Popliteal cyst	Eich et al., [35]	Knee: 33.3% detectable at 1 week and 1 month (no significance value given)	One patient experienced focal cutaneous atrophy and a possible ruptured popliteal cyst	Inconclusive due to lack of studies	
Destruction of articular cartilage/bone	Eich et al., [35]	Missing outcome data at 1 week and 1 month (no significance value given)	One patient experienced focal cutaneous atrophy and a possible ruptured popliteal cyst	Inconclusive due to lack of studies	
Destruction of meniscus	Eich et al., [35]	100% (no ligament destruction reported) at 1 week and 1 month (no significance value given)	One patient experienced focal cutaneous atrophy and a possible ruptured popliteal cyst	Inconclusive due to lack of studies	
Bone marrow oedema	Eich et al., [35]	None reported at 1 week and 1 month (no significance value given)	One patient experienced focal cutaneous atrophy and a possible ruptured popliteal cyst	Inconclusive due to lack of studies	
Avascular necrosis	Eich et al., [35]	None reported at 1 week and 1 month (no significance value given)	One patient experienced focal cutaneous atrophy and a possible ruptured popliteal cyst	Inconclusive due to lack of studies	
Uptake of contrast medium	Huppertz et al, [38]	Knee and Ankle: 20.0% detectable at 7 and 13 weeks (no significance value given)	Not Specified.	Inconclusive due to lack of studies	
US imaging					Weak evidence for IACIs decreasing US detectable clinical signs and symptoms as 2 studies show detectable improvement
Joint effusion and/or pannus	Eich et al., [35]	Knee: 100% detectable after 1 week and 1 month (no significance value given)	One patient experienced focal cutaneous atrophy and a possible ruptured popliteal cyst	Inconclusive due to lack of studies	
Popliteal cyst	Eich et al., [35]	Knee: 0% detectable after 1 week and 1 month (no significance value given)	One patient experienced focal cutaneous atrophy and a possible ruptured popliteal cyst	Inconclusive due to lack of studies	
Synovial hypertrophy	Laurell et al., [24]	Talocrural joint = 87% regression vs. 13% no effect	Local subcutaneous atrophy in 3 patients at 4 injection sites.	Inconclusive due to lack of studies	
		Posterior-STJ = 95% regression vs. 5% no effect			
		Midfoot joints = 91% regression vs. 9% no effect (no significance value given).			
		Mean synovial thickness: statistically significant difference at 4 weeks (p < 0.001)			
Synovial hyperaemia	Laurell et al., [24]	Talocrural Joint = 86% normalisation vs. 14% no normalisation	Local subcutaneous atrophy in 3 patients at 4 injection sites.	Inconclusive due to lack of studies	
		Posterior-STJ = 95% normalisation vs. 5% no normalisation			
		Midfoot Joints = 80% normalisation vs. 20% no normalisation (no significance value given) after 4 weeks.			

*Abbreviations:STJ* subtalar joint, *SD* standard deviation, *IACI(s)* intra-articular corticosteroid injection(s), *TH* triamcinolone hexacetonide, *TA* triamcinolone acetonide, *MP* methylprednisolone acetate, *BM* betamethasone, *MRI* magnetic resonance imaging *US* ultrasound, *IQR* inter-quartile range, *ROM* range of motion, *LLD* leg length discrepancy, *MTPJ* metatarsophalangeal joint, *IPJ* interphalangeal joint.

### Tenderness/pain

Three observational studies investigated measures of tenderness/pain in the lower leg joints, inclusive of the knee and ankle [24,35,38]. While all studies showed a reduction in pain after utilising IACIs, no significance values were given [24,35,38]. A weak level of evidence was found for IACIs decreasing lower leg tenderness/pain. Weak evidence for IACIs decreasing tenderness/pain was also found specifically in the knee (2 studies) and inconclusive evidence in the ankle due to the lack of studies (1 study).

### Swelling

Two observational studies investigated a reduction of swelling in the knee [35,38]. While no significance values were reported, there was weak evidence of IACIs decreasing swelling.

### Synovitis

Three observational studies investigated a reduction of synovitis in lower limb joints inclusive of the knee ankle, STJ, MTPJ and IPJ [40-42]. Inter-quartile ranges (IQR) were only available from one study reporting the overall mean relapse time of 0.5 yrs (IQR 0.3-1.3 yrs) compared with the mean remission time of 0.9 yrs (IQR 0.6-1.9 yrs) [40]. No significance values were given for the other studies [41,42]. A weak level of evidence was found for IACIs decreasing synovitis in lower leg joints. Specifically, a weak level of evidence was found for decreasing synovitis in the knee, ankle and STJ as two studies showed a reduction. There was inconclusive evidence for all other lower limb joints including MTPJ and IPJ.

### Effusion

Only one observational study investigated a reduction of effusion in the knee [38] and no significance values were given. There was inconclusive evidence for IACIs decreasing synovitis in the knee due to lack of studies.

### Hyperthermia

Only one observational study investigated a reduction of hyperthermia in the knee [35] and no significance values were given. There was inconclusive evidence for IACIs decreasing hyperthermia in the knee due to lack of studies.

### Sustained clinical response

Thirteen observational studies investigated a sustained clinical response post-injection in the lower leg joints, inclusive of the knee, ankle and foot [19,24,26,31-34,36,37,39,42,44,46]. Sustained clinical response included measures of pain, oedema, erythema and general inflammation, and was determined over periods ranging from 1-66 months post-injection, depending on joints investigated. Four studies showed statistically significant sustained clinical response [26,34,37,46]. Nine studies also found sustained clinical response in relation to duration of improvement and reduction/resolution of pain, inflammation, limitation of ROM, joint tenderness, swelling, temperature, joint effusion, deformity, synovitis, contracture and gait abnormalities. However, these results were derived from descriptive statistical analyses only [19,24,31-33,36,39,41,44]. Two studies found statistically significant sustained clinical response in the knee [34,37]. Six studies also found sustained clinical response in the knee. However, no significance values were given as data was presented using descriptive statistics only [31-33,36,39,44]. One study found statistical significance for the knee and ankle collectively [46]. Sustained clinical response was also found in three studies for the ankle [19,24,42], three studies for the STJ [19,26,42], and one study for the midfoot [19]. However, no significance values were given. Overall, there was weak evidence for IACIs decreasing clinical signs and symptoms in lower leg joints as thirteen studies showed a sustained clinical response post-injection.

### Joint ranges of motion

One RCT and four observational studies investigated joint ROM post-injection. The efficacy of IACIs was investigated for the STJ [25], knee [30,35,38] and ankle [24]. However, an odds ratio was only reported by one study for a mean duration of improvement of 1.2 yrs (SD ±0.9 yrs) [25]. Weak evidence was shown for IACIs increasing ROM in lower leg joints collectively. The RCT showed an increase in knee flexion following administrations of triamcinolone hexacetonide (TH) and betamethasone (BM) [30]. TH increased knee flexion more than BM [43]. Two observational studies also showed improvement of knee ROM [35,38] and weak evidence was found for IACIs increasing ROM in the knee. One observational study showed increased foot inversion and eversion [25] and ankle joint ROM normalisation/partial improvement [24]. Overall, there was inconclusive evidence for IACIs improving joint ROM in the STJ and ankle.

### Leg length discrepancy

One RCT and two observational studies investigated length discrepancies (LLD) [30,43,45]. Two studies specifically investigated LLD [43,45]. While both studies showed a decrease in the differences between limb length, only one was tested statistically and achieved statistical significance [43]. The other study only used descriptive statistics [45]. A weak level of evidence was found for decrease in lower LLD. One study also found a decrease in knee joint circumference; however, this was not statistically tested [30]. This particular study demonstrated that there was a decrease in knee joint circumference with use of TH or BM [30] but TH had a greater reduction compared to BM. Overall, there was inconclusive evidence for IACIs reducing knee circumference due to an insufficient number of studies.

### Disease activity outcomes detected by imaging

Four observational studies investigated the ability of imaging techniques to detect the efficacy of IACIs on JIA disease activity [24,35,38,42]. These studies showed IACIs led to clinical effects on disease activity as detected by imaging techniques when compared to clinical assessment. However, these differences were not compared using inferential statistics. Overall, there was weak evidence for the use of imaging techniques detecting the effects of IACIs. Specifically, two studies investigated the effects of IACIs using MRI to effusion [35,38]. Three studies investigated the effects of IACIs using MRI to detect pannus [31,34,44]. There was weak evidence for IACIs decreasing MRI detectable effusion and pannus. One study investigated MRI for detecting the effects of IACIs reducing popliteal cysts, destruction of articular cartilage/bone, destruction of meniscus, bone marrow oedema and avascular necrosis [35]. One study investigated MRI for detecting the effects of IACIs reducing the uptake of contrast medium [38]. However, there was inconclusive evidence for these subdomains due to a lack of studies. Overall, the level of evidence was weak due to two studies showing the effects of IACIs using MRI.

Two studies investigated the effects of IACS with musculoskeletal ultrasound imaging [24,35]. One study found decreases in signs of joint effusion and/or pannus and popliteal cyst [24], and one study found decreases in signs of synovial hypertrophy and synovial hyperaemia [35]. Therefore, the level of evidence assigned was weak for IACIs decreasing ultrasound detectable signs and symptoms, and inconclusive evidence for each specific subdomain.

## Discussion

Weak levels of evidence were found for IACIs decreasing tenderness/pain, swelling, synovitis, clinical signs and symptoms, increasing joint (ROM) in lower limb joints and medical imaging disease-related outcomes such as joint effusion, synovial hypertrophy and erosion. Inconclusive evidence was present for IACIs reducing effusion, hyperthermia, LLD and knee circumference. The majority of studies were low quality observational studies with one low quality RCT, and as such these conclusions are based upon weak evidence only. Additionally, the majority of the studies identified by the search strategy presented results using descriptive statistics only, leading to uncertainty regarding the effectiveness of IACIs versus a suitable comparator.

The results of this contemporary review demonstrate similar issues raised by previous reviews concerning the lack of conclusive evidence in support of IACIs improving clinical outcomes [16,17]. The adopted methodology of the most relevant previous systematic review appeared to be logical in restricting the inclusion criteria to RCTs in an attempt to increase the quality of evidence retrieved. However, at the time, there was a lack of published RCTs available for inclusion in this review and it subsequently concluded there was insufficient research to ascertain the effectiveness of IACIs for JIA. Observational studies were not considered by the authors as part of the inclusion criteria for that review, and as such potentially important studies were excluded which may have provided at least a weak level of evidence concerning IACI efficacy. In contrast, our review included observational studies and found weak evidence to suggest that sustained clinical response and improved joint ROM can be achieved following administration of IACIs to the joints of the lower limb in children with JIA.

A narrative literature review which adopted systematic search criteria on all medical treatment modalities for JIA including IACIs has previously been conducted [10]. The authors concluded there was significant evidence of efficacy for the use of IACIs in persistent oligoarticular JIA and moderate efficacy of IACIs for the use in polyarticular JIA based upon the findings of three studies [30,46,47]. However, this was not a true systematic review as the appraisal strategy did not adopt rigorous methods of quality assessment or evidence grading, and their conclusions were based solely upon effect sizes greater than 50% of patients. The lack of evidence grading introduces ambiguity to the conclusion of such reviews.

Our systematic review has utilised a specific quality assessment criteria with emphasis on the internal and external validity of the included studies [18]. Our study showed that while there was only one observational study with high internal validity [42], nine observational studies showed high external validity [19,24,26,32,34,35,40,44,46]. This may be explained by the fact that recruitment was largely conducted alongside standard practice within paediatric rheumatology outpatient clinics, thus increasing the clinical relevance of the results. This external validity criterion was also necessary to assess whether the study methodologies were pragmatic, as opposed to explanatory [48]. The pragmatic nature of these studies meant their conclusions were more likely to be applicable to the general population of children/adolescents with JIA to whom IACIs may be a possible treatment option.

Due to the lack of multiple RCTs in this area, no meta-analysis could be conducted. This has implications for future research as high quality RCTs and meta-analyses are necessary to inform evidence-based clinical practice. Indeed a pragmatic RCT of IACIs versus a suitable comparator for the relief of localised synovitis could have sufficient external validity to inform and influence current practice and health policy in future. However, it is recognised that several clinical practice guidelines recommend use of IACIs in spite of the lack of high quality research evidence supporting their use [10,20]. As such, it is acknowledged that a placebo- or sham-control arm for a parallel trial of IACIs may not be possible due to ethical restraints surrounding the withholding of standard care. A contextual problem with the lack of robust RCTs to evaluate IACIs at present exists with regards to recent changes to the Australian Medicare Benefits Schedule [49]. Evaluations of the cost-effectiveness of interventions are normally embedded within RCTs. At present, and to the best of the authors’ knowledge, an evaluation of the cost-effectiveness of IACIs in JIA has yet to be conducted. This raises an important issue; because presently people with arthritic conditions in Australia are potentially required to self-fund the cost of each individual IACI they receive [49], and doctors cannot yet provide sufficient guidance regarding this treatment based on robust evidence of clinical- and cost-effectiveness.

Two studies are frequently cited in reviews of IACIs in JIA and clinical practice guidelines to draw conclusions concerning this intervention [46,47]. These prospective, randomised studies focus on the comparative efficacy of commonly used steroid preparations triamcinolone hexacetonide (TH) and triamcinolone acetonide (TA), concluding that TH was superior. However, a major limitation of these studies was that a non-treatment, placebo, or sham control arm by which to compare the effects of the IACI interventions was not possible due to ethical constraints. Moreover, participants included were not on stable medication for an appropriate period prior to enrolment, so both studies were vulnerable to confounding bias. New evidence has emerged since previous reviews have been published; however, limitations in these studies are still evident. The well documented restrictions of the commercial availability of TH resulted in this preparation being replaced by TA in at least one study included in this review [25]. A lack of standardisation of IACI interventions was identified amongst studies retrieved by our search criteria which may be problematic. Frequently studies do not describe their injection technique, which can vary between individual institutes [22]. Moreover, studies included in this review have utilised varying post-injection management strategies including physiotherapy rehabilitation [31], immobilisation [31,34], and a period of non-weight-bearing or avoidance of physical activity [26,37,40,45]. Despite this, other studies included did not describe a post-injection strategy [24,35,38,42,43]. These approaches appear to lack consensus, and at present there is a lack of evidence available to support a specific post-injection procedure.

A novel finding from this review was that several studies employed image-guidance to aid their IACIs [24-26,38,42]. Ultrasonography-guided injections also have been identified as a useful imaging technique for IACIs as they permit the real-time guidance of the needle and allow confirmation of correct deposition of steroid [24,35]. These studies concluded the use of imaging techniques ensured correct needle placement and guided IACIs were superior to standard ‘blinded’ injection and aspiration techniques. These studies also raise further questions surrounding image-guidance concerning whether or not image-guidance can reduce the rate of complications associated with IACIs. At present there have been no RCTs comparing guided and blindly administered IACIs in JIA.

The conclusions from this review contribute to the evidence base in support of current JIA management practices through aggressive and early intervention to reduce disease activity [11]. This highlights inconsistencies and evidence gaps concerning the efficacy of IACIs in JIA. It also provides valuable additional and contemporary information through a robust and systematic approach relative to previous reviews. While investigating the efficacy of IACIs, post-injection procedure was included in the study description as a contextual factor. This did not occur in previous reviews. Not all studies included a post-injection technique. Those studies that did include post injection procedure are described in Table 3. However, the lack of consistency among the described injection procedures makes it difficult for comparison to make a consensus. This is an area which requires further research.

There were a number of limitations for this systematic review that should be acknowledged. Language was limited to English which is generally not recommended, but is often difficult to overcome [50]. There were two identified studies which had to be excluded based on language barriers (Spanish and Russian languages) that could have potentially had an impact on the findings of this review. It is acknowledged that a number of studies were identified by hand-searching that were not identified using the detailed search strategy. The original search strategy was reviewed to investigate whether or not it was adequate. The minor problem with the search strategy was subsequently attributed to an indexing issue within the databases that could not be overcome by altering the search strategy. However, in spite of this limitation, the pragmatic nature of the reviewing strategy permitted important and relevant studies which met the inclusion criteria to be identified by hand.

## Conclusions

In conclusion, there is conflicting and inconclusive evidence for the efficacy of lower limb IACIs for JIA. While there is enough quantity among the current body of knowledge to provide weak evidence in some outcome domains for generalised efficacy of IACIs administered to the lower limb, efficacy for duration of relief of synovitis in specific lower limb joints remains inconclusive. It is for this reason we conclude that more high quality studies are needed to definitively determine the efficacy of lower limb IACIs for improving outcomes associated with the JIA disease process.

## Competing interests

The authors have no competing interests to declare.

## Authors’ contributions

GJH and HJ were responsible for the conception of the study and all authors contributed to its design. HJ and KH were responsible for the acquisition and interpretation of the data with contributions from GJH where necessary. All authors were involved in drafting the article or revising it critically for important intellectual content and all authors approved the final version to be published.

## Supplementary Material

Additional file 1 Search strategy. Click here for file
