# Supplementary material for: The clinical effectiveness of intra-articular corticosteroids for arthritis of the lower limb in juvenile idiopathic arthritis: a systematic review
Source: Pediatr Rheumatol Online J. 2014 Jun 11;12:23. doi: 10.1186/1546-0096-12-23 (PMC4066295; doi:10.1186/1546-0096-12-23)
Supplement: Additional file 1 — Search strategy. [file 1546-0096-12-23-S1.docx]

**SUPPLEMENTARY APPENDIX A: SEARCH STRATEGY**

‘Juvenile idiopathic arthritis’ **OR** ‘Juvenile rheumatoid arthritis’ **OR** ‘Juvenile chronic arthritis’ **OR** (MeSH ‘Arthritis, Juvenile Rheumatoid’) **OR** ‘JIA’ **AND**

Lower* limb* **OR** (MeSH ‘Lower Extremity’) **OR** ‘lower extremity’ **OR** (MeSH ‘foot’) **OR** (MeSH ‘Foot joints’) **OR** ‘foot joints’ **OR** (MeSH ‘Knee’) **OR** ‘knee’ **OR** (MeSH ‘knee joint’) **OR** ‘knee joint’ **OR** (MeSH ‘leg’) **OR** ‘leg’ **OR** (MeSH ‘ankle’) **OR** ‘ankle’ **OR** (MeSH ‘ankle joint’) **OR** ‘rearfoot’ **OR** ‘hindfoot’ **OR** ‘midfoot’ **OR** (MeSH Forefoot, human’) **OR** ‘forefoot’ **OR** (MeSH ‘Subtalar joint’) **OR** ‘subtalar’ **OR** ‘talocrural’ **OR** ‘tibiofibular’ **OR** ‘talocalcaneal’ **OR** ‘calcaneocuboid’ **OR** ‘cuneonavicular’ **OR** ‘cuboideonavicular’ **OR** ’intercuneiform’ **OR** ‘tarsometatarsal’ **OR** ‘intermetatarsal’ **OR** (MeSH ‘Metatarsophalangeal joint’) **OR** ‘metatarsohalangeal’ **OR** (MeSH ‘toe joint’) **OR** ‘interphalangeal’ **OR** ‘talocuboid’ **OR** ‘cuneocuboid’ **OR** (MeSH ‘patellofemoral joint’) **OR** ‘patellofemoral joint’ **OR** ‘tibiofemoral’ **AND**

(MeSH ‘Injections, intra-articular’) OR ‘intra-articular injection*’ **OR** ‘intra articular injection*’ **OR** ‘joint injection’ **OR** ‘*steroid*’ **OR** ‘corticosteroid’ **OR** (MeSH ‘glucocorticoids’) **OR** ‘glucocorticoid’ **OR** ‘hydrocortisone acetate’ **OR** ‘methylprednisolone acetate’ **OR** ‘triamcinolone hexacetonide’ **OR** (MeSH ‘triaminolone’) **OR** ‘triamcinolone’ **OR** (MeSH ‘triamcinolone acetonide’) **OR** ‘triamcinolone acetonide’.
